# Isolation and complete nucleotide sequence of a Batai virus strain in Inner Mongolia, China

**DOI:** 10.1186/1743-422X-11-138

**Published:** 2014-08-06

**Authors:** Hao Liu, Xi-qun Shao, Bo Hu, Jian-jun Zhao, Lei Zhang, Hai-ling Zhang, Xue Bai, Run-xiang Zhang, Deng-yun Niu, Yan-gang Sun, Xi-jun Yan

**Affiliations:** 1 Division of Zoonoses, Institute of Special Economic Animal and Plant Sciences, Chinese Academy of Agricultural Sciences CAAS, 15 Luming Street, Jilin 132109, China; 2 College of Animal Science and Technology, Qingdao Agricultural University, Qingdao 266109, China

**Keywords:** Batai virus, Orthobunyavirus, Bunyaviridae, Reassortment

## Abstract

**Background:**

Batai virus (BATV) is a member of the *Orthobunyavirus* genus of the family Bunyaviridae, and a vector-borne pathogen. Genomic variations of BATV exist in different regions of the world, due to genetic reassortment. Whole-genome sequencing of any isolate is necessary for a phylogenetic analysis. In 1998, a BATV strain was isolated from an *Anopheles philippines* mosquito in Yunnan Province, China. This strain has not been found to infect any other host. We investigated BATV infection in cattle in Inner Mongolia, China and performed deep sequencing of the genome of the BATV isolate.

**Findings:**

Ninety-five blood samples were collected from cattle in Inner Mongolia, China in 2012. The BATV infection rate was 2.1%. Previously, BATV strain NM/12 was isolated from two cattle in Inner Mongolia, China, and the whole genomic sequence of the strain has been available. We determined the complete genomic nucleotide sequences of the small (S), medium (M), and large (L) genome segments using bovine blood obtained in 2012, and the nucleotide homologies of these segments with those from GenBank were 88.7%-97%, 84%-95.4%, and 72.6%-95.8%, respectively. The deduced amino acid identities were 87.2-99.7%, 64.2-96.8%, and 81.1-98.6%. Phylogenetic analyses based on full-length genomic sequences indicated that the M and L segments, and a portion of the S segment, of NM/12 are most closely related to the BATV strains isolated in Asia. The S and M segments of NM/12 were independent of phylogenetic lineages. The L segment was the most closely related to Chittoor/IG-20217 (isolated in India), and distantly related to isolated strains in Italy. Nucleotide substitution rates in the nucleotide sequences that code for the nucleocapsid, envelope glycoprotein, and polymerase protein of NM/12 strain were 2.56%, 4.69%, and 4.21%, respectively, relative to the original strain of MM2222.

**Conclusion:**

A novel BATV NM/12 strain from bovine serum collected in Inner Mongolia was isolated from cattle in China for the first time. Our findings elucidate the evolutionary status of the BATV NM/12 strain among different orthobunyavirus strains and may provide some clues to prevent the emergence of BATV infection in cattle and humans.

## Introduction

Batai virus (BATV) is a member of the *Orthobunyavirus* genus of the family Bunyaviridae. BATV was originally isolated from *Culex* mosquitoes in Malaysia in 1955 [1]. Serological surveillance and virus isolation have shown that BATV is widely distributed worldwide. Like several other orthobunyaviruses, BATV is an etiological agent of human and animal diseases. In humans, it can cause several clinical signs, including a febrile disease, and in ruminants it has been associated with a high incidence of abortions, premature births, and congenital defects [2,3]. It is also transmitted to humans and livestock by mosquitoes, biting midges (*Culicoides* spp.), and ticks, from frigid to tropical zones of Africa, Asia, and Europe.

Ngari virus has been identified as a naturally occurring reassortment between the BATV medium (M) segment and the Bunyamwera virus (BUNV) small (S) and large (L) segments. Intriguingly, this reassortment event is associated with increased virulence. This emphasizes the need for full-length characterization of all three genomic segments of bunyaviruses and an understanding of the pathogenicity in susceptible animals to better identify newly emerging viruses with potential significance for human and animal health.

The BATV genome is a single-stranded RNA comprising the S, M, and L segments, which respectively encode the nucleocapsid, envelope glycoprotein, and polymerase protein. Species of *Orthobunyavirus* are able to increase their genetic diversity through reassortment of genome segments during mixed infections [2]. BATV may also participate in genome segment reassortments with other viruses, which may produce new viruses with higher virulence. For example, Ngari virus is a genetic reassortment containing the M segment from BATV and the S and L segments from Bunyamwera virus [4].

Groseth et al. reported the full length sequences of MM2222, Chittoor/IG-20217, UgMP-6830, and MS50 strains of BATV isolated from Asian countries [5]. The nucleotide homologies of complete genomic sequences among BATV strains in Japan, Malaysia, India, and other Asian countries is 88.7%-97.9%, which is higher than that between Asia and European countries (84.7%-91.9%). The status of BATV as a vector-borne pathogen, its propensity for genetic reassortment, and its genomic variation in different parts of the world suggests that there may be much to be gained from whole-genome sequencing of isolates. Currently genomic analysis has been performed on the BATV strain YN92-4, isolated from an *Anopheles philippines* mosquito found in Yunnan Province, China in 1998 [6]. However, BATV infection has not been reported in other hosts in China.

Inner Monglolia where the BATV NM/12 strain was recently isolated from cattle is a remote province far from Yunnan province, China. Furthermore, the two strains (YN92-4 and NM/12) in China were isolated from the two different hosts (*Anopheles philippines* and cattle). However, the complete genomic sequence of these emerging strains is lacking in the literature, and the homology between the two isolates and whether any reassortment exists are unknown. In the present study, we investigated the prevalence of BATV infection in cattle in Inner Mongolia, China and performed deep sequencing of the genome of the BATV isolate.

## Materials and methods

### Case description and sample collection

During the course of surveillance for arboviral diseases in domestic animals, bunyavirus-like viruses were isolated in Inner Mongolia, China (110°46′-112°10′N, 40°51′-41°8′E). Infected cattle were initially mildly febrile, with loss of appetite and difficulty in maintaining balance. Ninety-five blood samples were collected from sentinel cattle in herds scattered across different locations in Inner Mongolia in 2012. No virus-causing illness was reported among these cattle.

### Virus proliferation

The hepatized blood was centrifuged and separated into plasma and blood cells. The blood cells were washed with phosphate-buffered saline and the plasma and blood cells were resuspended in PBS and stored at -80°C until virus isolation.

A litter of suckling mice (n = 15) was divided into three groups (n = 5 each). Five mice in one group were inoculated intracerebrally with 20 μL of serum suspensions in brain. The other five mice from the same litter were inoculated intracerebrally with Dulbecco’s modified Eagle’s medium supplemented with 2% fetal bovine serum as controls. The mice inoculated with the suspensions made from the cattle serum showed apparent paralysis with a lack of appetite on day 3 post-inoculation when all animals, including the healthy control mice, were euthanized. Brain tissues were collected from the paralyzed or control mice and tissues from the respective groups were pooled. The animal experimental protocols were approved by the Institutional Animal Care and Use Committee (IACUC) of the Chinese Academy of Military Medical Science, Changchun, China (10ZDGG007).

Brain tissue suspension (20 μL) was propagated in Vero cells in complete minimum essential medium supplemented with 5% fetal calf serum and 1% penicillin (10,000 U/mL)/streptomycin (10,000 μg/mL) at 37°C in a 5% CO_2_ environment for 72 h. The cytopathogenic effects were observed in the Vero cell cultures inoculated with the brain tissue suspensions of suckling mice. The cells with cytopathogenic effects (CPEs) were frozen/thawed three times and the supernatant was collected and inoculated vero cells again. The plaques were picked out. The above process was repeated for approximately ten rounds until enough virus was amplified. The supernatants containing BATV was resuspended and examined under a transmission electron microscope (TEM) after negative staining.

### Animal challenge test

Twenty-five healthy domestic suckling mice and three-week-old mice free of BATV antibodies were divided into five groups. Samples (0.02 or 0.05 mL) containing 10^5^ of 50% tissue culture infective dose (TCID_50_) of the NM/12 strain was inoculated into brain of the suckling and 3-week-old mice, with mock inoculated phosphate buffered saline (PBS) as the control group. Clinical signs of each mouse were observed daily. From the fifth day post-inoculation, samples of dead suckling mice and serum suspensions from three-week-old mice were collected for polymerase chain reaction (PCR) with P1 and P2 primers to detect BATV.

### Isolation of viral RNA

To identify the isolated viruses, total RNA was extracted from supernatants of the serum suspensions of cattle, suckling mice, and cell culture that showed CPEs using a TaKaRa MiniBEST Viral RNA/DNA Extraction Kit (TaKaRa China, Dalian, P.R. China) in accordance with the manufacturer’s instructions. The denatured RNA was incubated at 42°C for 50 min to perform first-strand cDNA synthesis using reverse-transcription (RT)-PCR with random primers, as previously described [7].

### Nucleotide sequencing of the BATV NM/12 genomes

To detect BATV in the suspensions of cattle, suckling mice, and cell culture, PCR to amplify the M gene with M gene-specific primers (Table 1) was performed using the virus RNA from the above as template. All sequences were synthesized at Shanghai Sangon Biological Engineering Technology and Service, Shanghai, China.

**Table 1:** Primers used to amplify and sequence the complete BATV S, M, and L segments

**Segment primer**	**Sequence (5′ to 3′)**	**Amplicon size (nt)**
S segment primer		
BATAS1F	5′-GAATTCAATGATGTCGCTGCT-3′	818
BATAS1R	5′-CAATTTGCTGTGCTCTTTCTG-3′	
3′S-Inner Primer	5′-CTAGCAAAGTTTGGCATCAGC-3′	
3′S-Outer Primer	5′-GGGTGGAAGAAGACAAATGTG-3′	
5′S-Inner Primer	5′-GGGTCAAAAGTACTGCTGGTG-3′	
5′S-Outer Primer	5′-GTGTAGATACGCTTAAAGTTA-3′	
M segment primer		
BATAM1F	5′-GATGTTGCTACTTCTTGTCTT-3′	2013
BATAM1R	5′-AGTTTTGTAATACCAGTTGTG-3′	
BATAM2F	5′-CAACTGAGTCACTCTTTTATG-3′	1973
BATAM2R	5′-CATAATCTTGTTTTGTGGAGG-3′	
BATAM3F	5′-CTGCAGTTTTAAGATTGTTTC-3′	480
BATAM3R	5′-TGTTTTATTTCCTGTAGGTAC-3′	
3′M-Inner Primer	5′-GTACCTACAGGAAATAAAACA-3′	
3′M-Outer Primer	5′-TGATGTTTAAACTTAGAGATG-3′	
5′M-Inner Primer	5′-GAGGCAAAATTCTGATATAGC-3	
5′M-Outer Primer	5′-ATTTTTTTCATATATTATTTC-3′	
L segment primer		
BATAL1F	5′-AAAAATGGATGATCAGATGTA-3′	2025
BATAL1R	5′-TTCTCTGCTATATAATCTTTG-3′	
BATAL2F	5′-TCGATATATGATAATGAATTC-3′	2021
BATAL2R	5′-CTAAACCAACTAAAGCTATCA-3′	
BATAL3F	5′-TTAAGAGTCGTCATGATATAC-3′	1952
BATAL3R	5′-TGATAATACAACAGGACAGAC-3′	
BATAL4F	5′-TAAAAAGAAATGAAGAAGGAC-3′	945
BATAL4R	5′-CTTAGAAAAAGGTGAACATGG-3′	
3′L-Inner Primer	5′-CAACTGGACCAAGATGCTAAA-3′	
3′L-Outer Primer	5′-CTGCCTTCTACATAAATACAG-3′	
5′L-Inner Primer	5′-GTTCCAACTGATACTTTATAG-3′	
5′L-Outer Primer	5′-GTATTTTTTATAAGTAATCTC-3′	
P1	5′-ATAGAATCAGCAATAGCAAGC-3′	550
P2	5′-ATGATGATCTGTAACCTCTAA-3′	

Sequences of oligonucleotide primers (Table 1) were designed according to the BATV Genbank genome sequences JX846603, AB257763, and JX846595 that coded for the nucleocapsid, envelope glycoprotein, and polymerase protein, respectively. The 3′ and 5′ termini of these viral genes were amplified by 3′ and 5′ rapid amplification of cDNA ends (RACE) [8,9]. PCR was carried out using 35 cycles of amplification. The presence of the correct PCR products was confirmed by electrophoresing 10 μL through 1.0% agarose gels. To achieve high-quality consensus sequences and to avoid laboratory PCR artifacts, each entire genome was sequenced at least three times. The amplified fragments were cloned into a pMD18-T vector and further confirmed by sequencing (Shanghai Biological Engineering, Shanghai, China). The full-length genome was compiled using the DNASTAR software program [10].

### Public data sets

Full-length genes of the S, M, and L segments from the NM/12 strain were deposited in the GenBank database under accession numbers KJ187038, KJ187039 and KJ187040.

### Multiple alignments and phylogenetic analyses

The BATV strains were used for multiple sequence alignments and phylogenetic analyses, with a description of the history of these strains and their GenBank accession numbers. Multiple sequence alignments and sequence similarity calculations between aligned nucleotide and amino acid sequences were performed using DNASTAR software. Multiple sequence alignments and phylogenetic trees were produced using MEGA 5.2 software and constructed from aligned nucleotide sequences using the neighbor-joining method. The stability of the tree obtained was established by bootstrapping analysis with 1000 replications [11].

## Results

### RT-PCR assays of clinical samples for two suspicious pathogens

**Figure 1:**
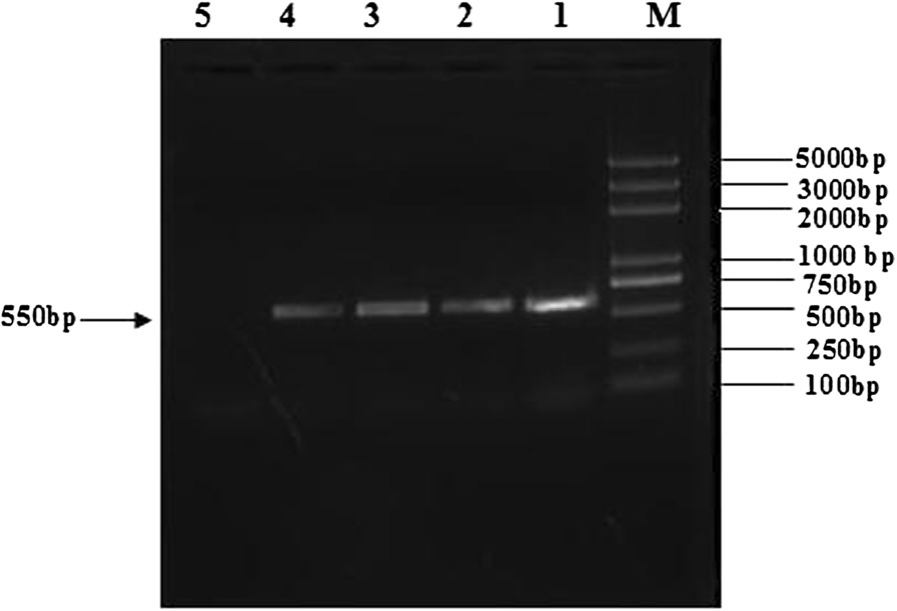
**Amplification of the M gene from BATV by RT-PCR with P1 and P2 primers.** BATV RNA was used as template and specific primers targeting the virus M gene were used in an RT-PCR assay. (Lanes: M, Trans 2K DNA Marker; Lanes: 1–2, blood samples of cattle; Lanes: 3, cytopathic effect of Vero cell; Lanes: 4, Death of the Challenged Mice; Lanes: 5, negative control).

An emergent BATV strain was isolated in Inner Mongolia, China. It was found that the BATV infection rate in cattle was 2.1% (2/95). The agarose gel electrophoresis results showed that a target fragment of 550 bp in length was amplified by RT-PCR, from RNA extracted from blood samples of the febrile cattle (Figure 1).

### Virus isolation and TEM examination of the cell cultures

**Figure 2:**
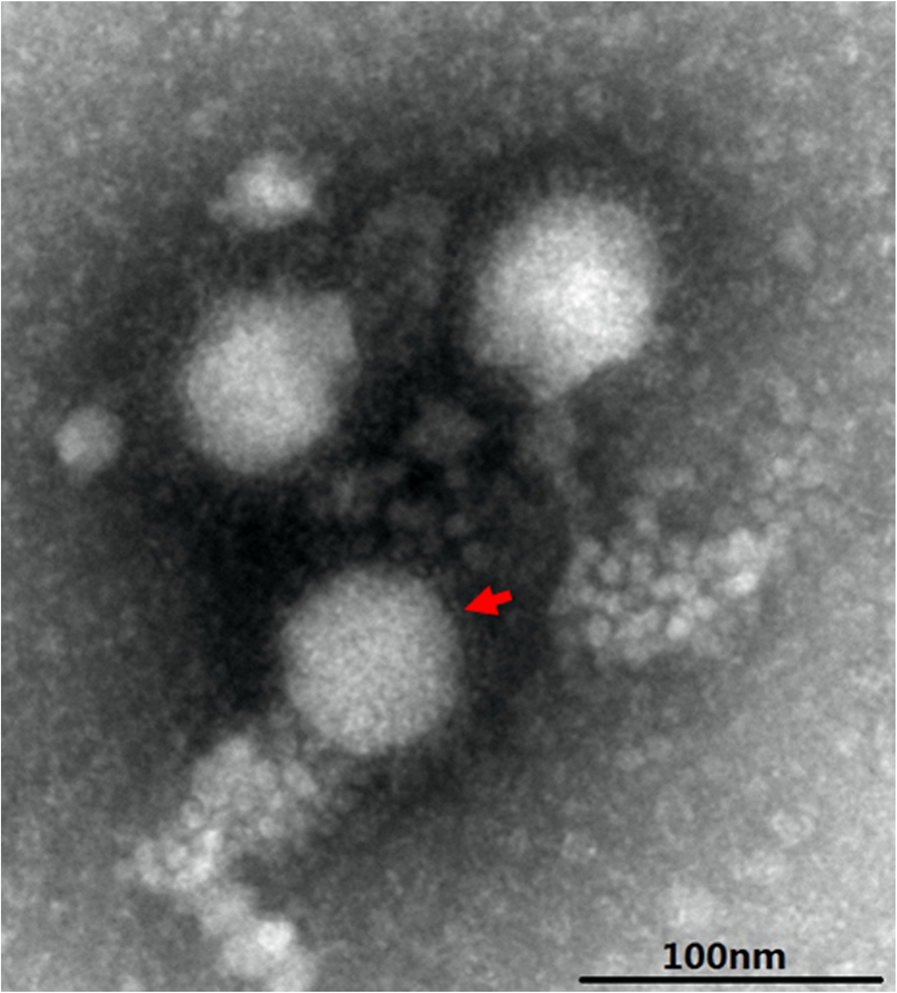
**Electron microscopy of negative-stained Batai virus particles from cattle.** Scale bar indicates 100 nm.

At 72 h post-inoculation the suspensions of febrile cattle brain tissue and Vero cell cultures showed a distinct CPE characterized by cell rounding, pyknosis, ‘Fleece-Pulling’ to the thyrsoid and cell monolayer destruction (data not shown). TEM of negatively stained samples revealed medium-sized particles with a diameter of approximately 50 nm within an envelope, and characteristics of typical of BATV (Figure 2). RT-PCR revealed that the supernatants of cultures were positive only for BATV (Figure 1). The positive PCR products of the M gene from the brain tissue suspensions and cell cultures were identified by sequence analysis. The 550-bp sequence of the M gene from BATV from the suspensions shared 100% homology with that of Vero isolates.

### Clinical symptoms of the challenged mice

At five-days post-inoculation, suckling mice began to die, and three-week-old mice showed lack of appetite, poor grooming, and difficulty in walking. After 10 and 18 days post-inoculation, all suckling mice died, but three-week-old mice recovered completely and exhibited no signs of infection. RT-PCR assays of the 550 bp sequence of the M gene from BATV from the death of mice were performed(Figure 1).

### Complete sequencing of the BATV NM/12 genomes

Nucleotide sequences of individual cDNA fragments and their junctions in the viral genome were obtained. The full-length genome of NM/12 consisted of a 947-nt S segment, a 4405-nt M segment, and a 6870-nt L segment and contained one open reading frame that encode three proteins of 151, 943, or 1395 amino acids (Table 2). The viral RNA segment S was 177 nt in the 5′noncoding region and 68 nt in 3′ noncoding region (in viral RNA orientation). Segment M had 93 and 42 nt in 5′ and 3′ noncoding regions, respectively. The L segment had 68 and 48 nt in 5′ and 3′ noncoding regions, respectively. Full-length genes of S, M, and L segments from the NM/12 strain were deposited in the GenBank database under accession numbers KJ187040, KJ187039, and KJ187038, respectively.

**Table 2:** Genome sequence analysis of the NM/12 strain of Batai virus (BATV)

	**Size**		**Nucleotide substitution**		**Amino acid substitution**	
**Genome segment**	**Nucleotides**	**Amino acids**	**No. of substitutions**	**% substitutions**	**No. of substitutions**	**% substitutions**
5′-NCR	177	0	6	5.13	0	0.0
S(CDS1) and (CDS2)	702 and 306	234 and 102	18 and 8	2.56 and 2.61	1 and 1	0.43 and 0.98
3′-NCR	68	0	0	0.0	0	0.0
5′-NCR	93	0	0	0.0	0	0.0
M(CDS)	4305	1435	202	4.69	44	3.06
3′-NCR	42	0	0	0.0	0	0.0
5′-NCR	68	0	3	4.41	0	0.0
L(CDS)	6714	2238	283	4.21	28	1.25
3′-NCR	48	0	0	0.0	0	0.0

The nucleotide sequence of the NM/12 genome was compared with that of the MM2222 strain.

NCR - non-coding region.

### Phylogenetic analysis based on three cloned gene fragments of BATV

**Figure 3:**
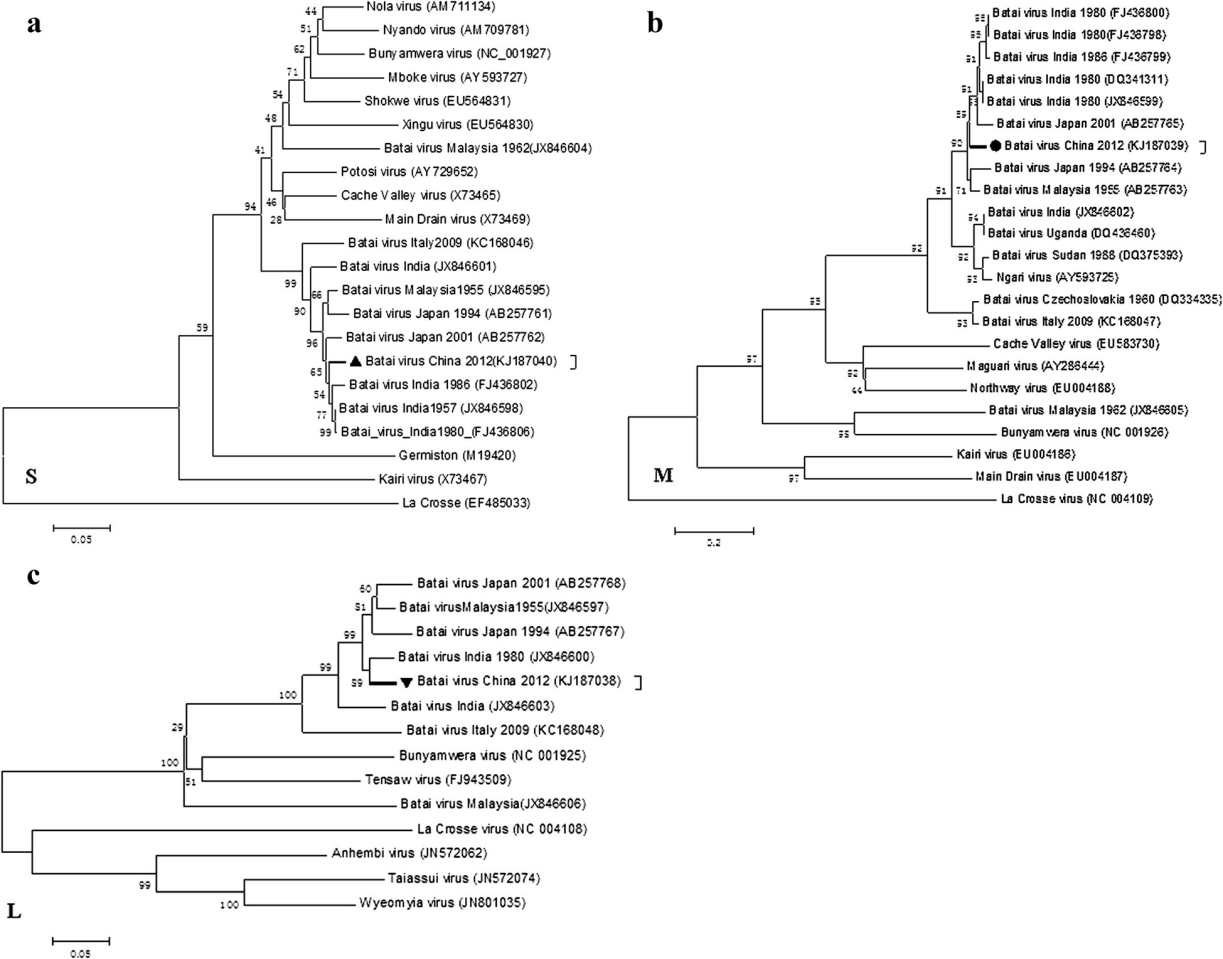
**Phylogenies of the S, M and L segments of the genus Orthobunyavirus.** The genome of BATV N and NSs genes of partial S segment (**a**; length =911 nucleotides), M (**b**; length = 4405 nucleotides), and the partial L segment (**c**; length = 580 nucleotides) representative Orthobunyavirus for the aligned sequence and phylogenetic tree.The phylogenetic tree was constructed by the neighbor-joining method using 1000 bootstrapping replicates.

The genome was compared with that of other orthobunyaviruses, namely, strains ON-7/B/01 (from Japan), UgMP-6830 (Uganda), Chittoor/IG-20217 (India), MM 2222 (Malaysia), MS50 (Malaysia), and other Batai viruses, to determine how they were related to other partially and fully-sequenced genomes of BATV strains previously reported (Figure 3a-c). The S, M, and L segments of NM/12 were most closely related to the BATV strains isolated in Asia. S and M segments of NM/12 strain were independent of phylogenetic lineages. The L segment was the most closely related to Chittoor/IG-20217 (isolated in India) and distantly related to strains isolated in Italy (Figure 3a-c). The sequence comparison showed that NM/12 had several nucleotide substitutions distributing throughout the genome.

In a previous report the 5′ and 3′ termini of each genomic segment were highly conserved among the strains in the above except NM/12 strain which has a few nucleotide substitutions. A total of 36, 202, and 283 nucleotide substitutions were found in S, M, and L segments of the NM/12 strain, respectively (Table 2). The full-length genome analysis indicates that the virus may not have undergone any reassortment. NM/12 shared 92.1% of the S nucleotides, 97.2% of the M, and 93.8% of the L with the original strain of MM2222, and the corresponding deduced amino acid sequence identities were 97.3% in the S, 95.4% in the M, and 95.8% in the L segments (Table 2). Two, 44, and 28 amino acid substitutions were found in the genome of NM/12. Nucleotide substitution rates were 2.56%, 4.49%, and 4.21% in the nucleotide sequences that code for the nucleocapsid, envelope glycoprotein, and polymerase protein, respectively (Table 2).

These findings indicate that there were variations in the S, M, and L gene coding regions in the NM/12 strain compared with the different other strains above. It was reported in a previous study that the YN92-4 strain is only close to the ON-7/B/01 strain isolated from cattle but distal to other strains [6]. NM/12 strains has shown great changes in nucleotide sequences in different hosts and regions compared to the YN92-4 strain in China. Therefore, the NM/12 strain may have a high potential for evolutional changes. It is possible that variations in the BATV genome may occur constantly to adapt to different environments.

## Discussion

The Batai virus has been isolated from mosquitoes, cattle, pigs, and febrile patients, and has been identified as a naturally occurring genetic reassortant. It is transmitted to humans and livestock by mosquitoes, biting midges (*Culicoides* spp.), and ticks, from frigid to tropical zones of Africa, Asia, and Europe. Despite the potential health risks posed by bunyavirus reassortment (for example, the recent emergence of Schmallenberg virus) [12] a lack of complete sequencing data for many of these viruses complicates surveillance and virus identification efforts. Full-length genome sequence data are available for very few orthobunyaviruses [5].

The genomic sequences of the BATV strains of Japan, Malaysia, India, and other Asian countries share more homologies compared with the strains of Asia and European countries. Yet, they do have genetic differences and a propensity for genetic reassortment. Reassortment between the BATV M segment and the BUNV S and L segments is associated with increased virulence [13,14]. In 1997 and 1998, M segment reassortment between the Batai and Ngari viruses was associated with severe febrile disease outbreaks in East Africa, and hundreds of thousands of people died from the disease [4]. Genomic reassortments between different viruses within the genus Orthobunyavirus, such as reassortment between the Tinaroo and Jatobal viruses of the Simbu serogroup [2], have also been previously observed. These findings emphasize the need for full-length characterization of all three genomic segments of the bunyaviruses to better identify newly emerging viruses with potential significance for human or animal health.

In China, the Batai virus was first isolated from an *Anopheles philippines* mosquito in Yunnan Province in 1998. Phylogenetic analyses based on the genomic sequences of the S, M, and L segments revealed that the YN92-4 strain isolated in China belongs to the same group as the MM 2222 strain isolated in Malaysia, but the complete genomic sequence has not been published.

In the present study, we first isolated a novel Batai virus NM/12 strain from bovine serum in the Inner Mongolian region of China and performed the complete genome sequencing of the emerging BATV NM/12 strain. This is the first report of a BATV strain isolated from cattle in China. To elucidate the phylogenic relationship of the NM/12 strain, we carried out multi-segment phylogenetic analyses of the NM/12 strain and all other strains in GenBank. We found that the NM/12 strain is closely related to strains in different regions of Asia and distantly related to European BATV strains. Based on partial sequencing, NM/12, ON-1/E/94, and ON-7/B/01 appeared in the same phylogenetic lineage but diverged at different times despite that both were isolated separately from bovine blood in China and Japan.

Our full-length genomic analysis indicated that the virus may not have undergone any reassortment. Homologies of the nucleotide (amino acid) sequences between the S, M, and L segments of NM/12 and the original MM 2222 strain were 92.1% (97.3%), 97.2% (95.4%), and 93.8% (95.8%). The sequence comparison showed that the NM/12 strains had a number of nucleotide substitutions that were scattered throughout the genome containing 5′-NCR. In addition, the 5′ NCR nucleotide substitution rate was significantly higher than that of the 3′ NCR. The open reading frames and untranslated region genomic segment were conserved among BATV [15]. Nucleotide substitution rates were 2.56%, 4.49%, and 4.21% in the parts of the genome that code for the nucleocapsid, envelope glycoprotein, and polymerase protein, respectively (Table 2).Pair-wise alignment of the complete S, M, and L segments of the BATV NM/12 strain with those of the strains in GenBank revealed the phylogenetic relatedness among these BATV strains (Figure 3a-c). Unlike the Ngari virus, the NM/12 strain had no genetic reassortment among all the compared viruses but was close to the Batai virus strains isolated in Asian countries, except Japan. The S and M segments of the NM/12 strain were close to ON-7/B/01 isolated from cattle serum in Japan, but they were in different phylogenetic lineages. Only the L segment was closely related to Chittoor/IG-20217 (isolated in India). This finding indicates that all the BATV strains have few variations, and no reassortment among the compared BATV strains was found. This was confirmed by the amino acid divergence shown through complete sequence analysis in this study. However, there are genomic reassortments between different viruses within the genus Orthobunyavirus, such as the reassortment between the Tinaroo and Jatobal viruses.

Batai virus isolated from mosquitoes, cattle, pigs, and febrile patients were identified as naturally occurring reassortants between the BATV M segment and the BUNV S and L segments. This reassortment is associated with increased virulence. BATV antibody was found in fever patients in Yunnan province, China after the BATV was isolated from *Anopheles philippines* in Yunnan Province in 1998 [13,14,16]. We found that suckling mice infected with the NM/12 strain had high morbidity, indicating that the NM/12 strain of BATV may also have strong infectivity in other vertebrate hosts. The findings in this study provide further insight into the genetic diversity of Orthobunyavirus and draw attention to the need for prevention of BATV infection in cattle and humans in China.

## Competing interests

The authors declare that they have no competing interests.

## Authors’ contributions

LH, YXJ, SXQ participated in the study design. LH, HB, ZJJ, ZRX carried out the immunoassays. ZL, BX, ZHL performed the sequence analysis, and all authors participated in writing and revising the manuscript. All authors have read and approved the final manuscript.
